# Complex Water Networks Visualized through Cryogenic Electron Microscopy of RNA

**DOI:** 10.1063/4.0001087

**Published:** 2025-10-27

**Authors:** Rachael C Kretsch, Shanshan Li, Grigore Pintilie, Michael Z Palo, David A Case, Rhiju Das, Kaming Zhang, Wah Chiu

**Affiliations:** 1 Stanford University, Palo Alto, CA, USA; 2 University of Science and Technology of China, Hefei, N/A, China; 3 Rutgers University, Piscataway, NJ, USA

## Abstract

The stability and function of biomolecules are directly influenced by their myriad interactions with water. Nucleic acids are highly solvated and hence uniquely suited for the investigation of water in near-native conditions using cryogenic electron microscopy (cryo-EM). We determined two cryo-EM maps of *Tetrahymena* ribozyme 2.2 and 2.3 Å resolutions and automatically modeled and cross-validated water molecules and Mg^2+^ ions in the ribozyme core, revealing the extensive involvement of water in mediating RNA non-canonical interactions. Unexpectedly, in regions where we do not model ordered water, we observed highly similar densities in both cryo-EM maps. In many of these regions, the cryo-EM densities superimpose with complex water networks predicted by molecular dynamics (MD), supporting their assignment as water and suggesting a biophysical explanation for their elusiveness to conventional atomic coordinate modeling. The cryo-EM density can be further compared against various MD simulations enabling evaluation and comparison of the accuracy of various simulation methodologies.

